# On the Use of Heterogeneous Stock Mice to Map Transcriptomes Associated With Excessive Ethanol Consumption

**DOI:** 10.3389/fpsyt.2021.725819

**Published:** 2021-10-12

**Authors:** Robert Hitzemann, Denesa R. Lockwood, Angela R. Ozburn, Tamara J. Phillips

**Affiliations:** ^1^Department of Behavioral Neuroscience and Portland Alcohol Research Center, Oregon Health & Science University, Portland, OR, United States; ^2^Veterans Affairs Portland Health Care System, Portland, OR, United States

**Keywords:** RNA-Seq—RNA sequencing, alcohol use disorder (AUD), genetic variability, gene networks, excessive ethanol consumption

## Abstract

We and many others have noted the advantages of using heterogeneous (HS) animals to map genes and gene networks associated with both behavioral and non-behavioral phenotypes. Importantly, genetically complex *Mus musculus* crosses provide substantially increased resolution to examine old and new relationships between gene expression and behavior. Here we report on data obtained from two HS populations: the HS/NPT derived from eight inbred laboratory mouse strains and the HS-CC derived from the eight collaborative cross inbred mouse strains that includes three wild-derived strains. Our work has focused on the genes and gene networks associated with risk for excessive ethanol consumption, individual variation in ethanol consumption and the consequences, including escalation, of long-term ethanol consumption. Background data on the development of HS mice is provided, including advantages for the detection of expression quantitative trait loci. Examples are also provided of using HS animals to probe the genes associated with ethanol preference and binge ethanol consumption.

## Introduction to HS Mice

McClearn and Rodgers (1) observed that among five inbred mouse strains there was a marked difference in ethanol preference (2-bottle choice, water vs. 10% ethanol). Of the strains tested, the C57BL/6 (B6) showed the highest preference. This experiment, with numerous variations, has been repeated hundreds of times [e.g., (2)] with the B6 strain consistently showing a high preference. Further, the B6 strain shows the highest binge ethanol consumption when tested in the Drinking-In the-Dark (DID) model (3). These data have cast a long shadow on ethanol research resulting in the almost exclusive use of the B6 strain to test for mechanisms of ethanol action and for new therapeutic treatments. This monoculture focus has some obvious advantages including replicability across laboratories and the ability to use genetically modified mice, which are almost exclusively on a B6 or largely B6 background, for hypothesis testing. These and related advantages are substantial. However, the major disadvantage of using the B6 strain or even B6 diallel crosses (e.g., B6 × DBA/2 [D2]) is that the biology extracted may not be generally applicable. Thus, important pathways are missed due to the lack of genetic diversity and further, individual variation, a key component of some analyses, will be substantially reduced. One solution to these problems is the use of outbred mice and heterogeneous stocks (HS) [see e.g., (4, 5)].

The first widely used mouse HS appears to be the HS/Ibg, described by McClearn et al. (6). This HS was a cross of 8 laboratory mouse strains; the cross was begun at Berkeley before the mice were transferred to the Institute for Behavioral Genetics (Boulder), hence the Ibg designation. For an 8-way cross there are >40,000 possible breeding funnels. Rather than dealing with this issue, the colony was formed balancing for the Y chromosome from each of the founder strains. The colony was maintained at ~40 families. These mice served as the founders for a number of alcohol-related selected lines including the Long Sleep/Short Sleep, Withdrawal Sensitive Prone(WSP)/Withdrawal Sensitive Resistant (WSR), the FAST/SLOW and the High Alcohol Preference/Low Alcohol Preference lines (7–10). Here, we briefly focus on the replicate WSP/WSR selected lines; the lines were selectively bred for withdrawal severity after cessation of 3 days of ethanol vapor exposure. Crabbe et al. (11) discussed the consilience of the mouse genetic models with human genetics in some detail. It was concluded that the overlap was greatest for tolerance and withdrawal and that for both mice and humans, these phenotypes had independent genetic risk. From the perspective of alcohol use disorder, the question naturally arises as to whether the WSP and WSR lines differ in ethanol consumption. Previous studies in animals with a B6xD2 genotype [see details in Metten et al. (12)] suggested a strong negative genetic relationship between withdrawal and consumption, although there are exceptions (13). In the WSP/WSR lines derived from HS founders, the situation appears more complex. Crabbe et al. (13) found, as predicted, the WSR-2 line had significantly higher preference than the WSP-2 line, but the opposite line difference was found for the WSP-1 and WSR-1 lines. Regarding drinking in the dark (DID), a model of binge consumption (see below), both WSP lines consumed more ethanol and had higher BECs than the WSR lines; thus, greater genetically-determined withdrawal severity predicted higher ethanol consumption, opposite to previous findings (12). Turning things around, the High DID-1 and−2 selected lines (selectively bred from HS/NPT founders—see below) do not differ in withdrawal severity after cessation of vapor inhalation. There are many interpretations of these data. However, we simply wish to make the point that lessons learned from simple crosses may not apply to HS and vice-versa.

**Figure 1:**
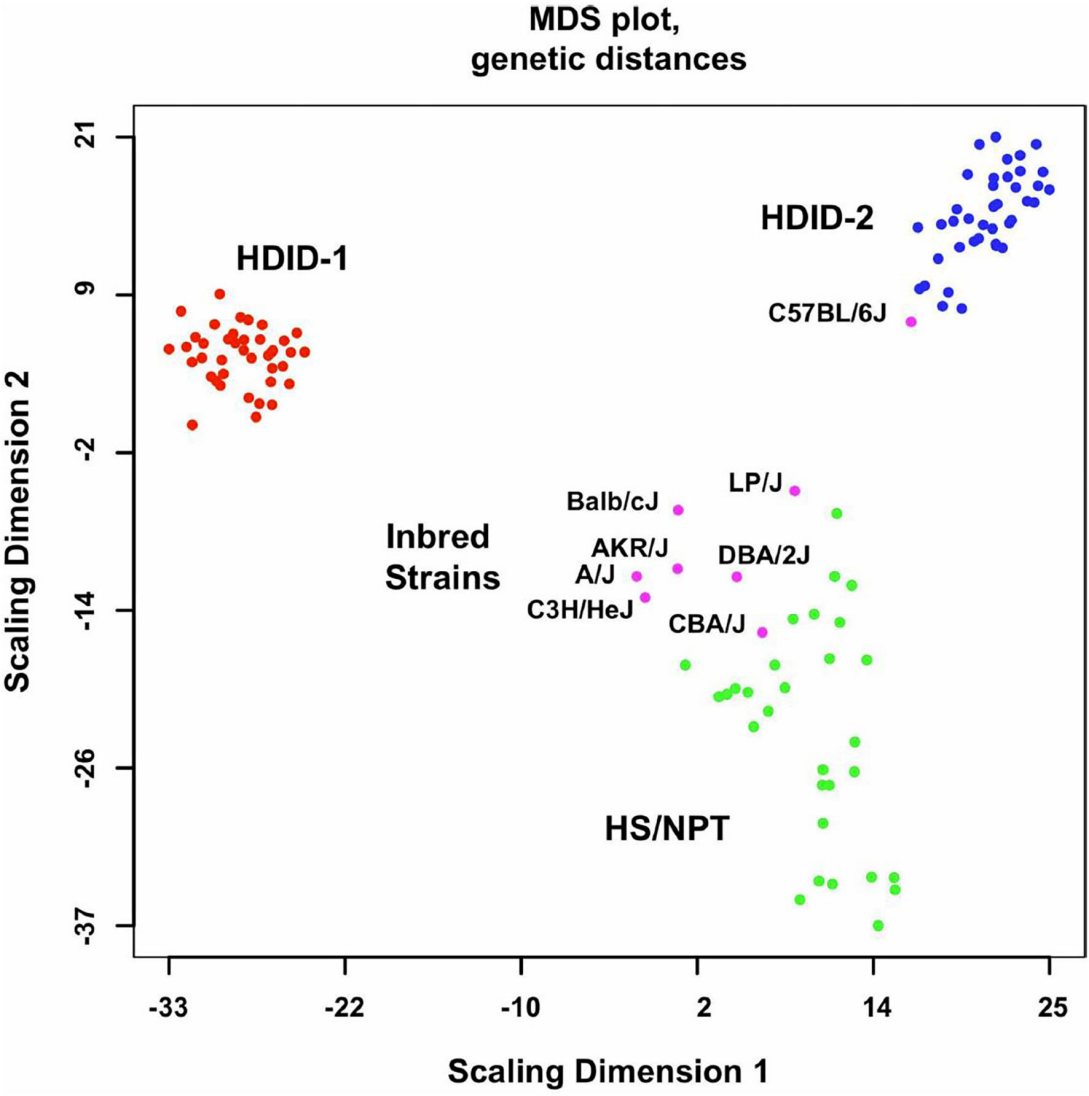
Genome-wide genetic distances between the HDID-1 and -2 selected lines, HS/NPT animals and the inbred strains used to form the HS/NPT. Details of the animals used are found in the Methods in (52). Data are presented as a multidimensional scaling (MDS) plot. Note the greater dispersion in the HS/NPT animals when compared to the HDID-1 and HDID-2 selected lines and the differences between the two selected lines. Also note that among the inbred strains the C57BL/6J is distinct from the other 7 founders. Figure reprinted with permission from (52).

In 1991, Gerry McClearn suggested to one of us (RH) that there was a need for a new HS. Two of the HS/Ibg founder strains (Is/Bi and RIII were no longer available for testing) and random genetic drift over the >25 years of breeding was likely to have significantly distorted allele frequencies. Our interest at the time was not in ethanol-related behaviors, but rather in haloperidol-induced catalepsy [see e.g., (14)] and in developing haloperidol response selected lines. The 6 HS/Ibg founders available for testing were skewed to very haloperidol responsive strains. Two non-responsive strains (CBA/J and LP/J) were chosen to fill out the 8 founders for developing a new HS. However, it should be noted that the 8 strains included 2 representatives each from 4 different phylogenetic clades [see Figure 1b in (3)]. The new HS was formed by pseudo-random breeding at the Northport VA, hence the NPT designation. The first report on the HS/NPT is found in Hitzemann et al. (15). For more than 25 years, the HS/NPT have been maintained as 48 families using a circle breeding design. HS/NPT were first used in ethanol research to fine map a QTL for ethanol-induced locomotor activation on chromosome 2 (16).

Breeding pairs from each of the 48 HS/NPT families were shipped in 2000 to Jonathan Flint (Oxford, U.K). Over the next several years >2,400 animals were phenotyped for a variety of physiological and behavioral traits (17). Valdar et al. (18) examined the genetic and environmental effects on 88 of these traits and mapped the QTLs for 97 traits to a reasonably high resolution (19). Huang et al. (20) mapped eQTLs in a subset of the tested animals; data were obtained for the hippocampus, liver and lung. Although these authors noted a large number of hybridization artifacts for detecting eQTLs, the data obtained remain an important feature in evaluating HS/NPT data. Of equal importance, the 8 HS/NPT founders were among the 17 strains initially sequenced as part of the Mouse Genomes Project (21).

Twenty years ago, members of the Complex Trait Consortium (CTC), later renamed the Complex Trait Community, began a series of meetings to develop the Collaborative Cross [CC] (22). The CC was proposed as a large panel of recombinant inbred (RI) strains derived from a genetically diverse HS. The initial plans were to develop more than 1,000 RI strains. Much of the early CC planning sessions focused on determining the 8 strains that would be crossed to form the HS founders. How the final 8 strains were chosen could easily be the subject of another review. There was however, general agreement that three wild-derived strains (WSB/EiJ, CAST/EiJ, and PWK/PhJ) would be included, which in turn would boost the overall HS genetic diversity to more than 90% of what is available in *Mus musculus* [see (23)]. In 2005, we began crossing the 8 CC founder strains. Thirty-two unique breeding funnels were used and each funnel was bred in duplicate [see (24) for breeding details]. Of the 64 breeding funnels, 3 produced no offspring, but each was unique. The 32 families were expanded to 48 and have been bred continuously since 2007 using a circle breeding design. This HS was designated the HS-CC (25).

The Diversity Outbred HS were formed by crossing 144 of the partially inbred CC lines [see (26) for breeding details]. The DO colony is maintained as a panel of ~175 breeding pairs; all matings are randomized with avoidance of sibling matings. The HS-CC and DO were compared in (27). Of particular note, a meiotic drive locus on chromosome 2 has been eliminated from the DO but not the HS-CC. However, this difference does not appear to have affected the high ethanol preference found in both the HS-CC and DO. Given the larger DO breeding population, genetic drift in the DO compared to the HS-CC will be slower. Compared to the HS/NPT, ethanol preference in the HS-CC and DO is 3–4 times higher. The reason for the higher preference in both populations would appear to be at least partially associated with the fact that in addition to the B6 strain, the PWK/PhJ founder strain also has a high ethanol preference (28).

## Transcriptomics in HS Populations

Sandberg et al. (29) were the first to detect differences in genome-wide brain gene expression between 2 inbred mouse strains (B6 and129S6/SvEvTac). Several differentially expressed (DE) genes aligned with known behavioral quantitative trait loci (bQTLs). For example, *Kcnj9* was DE and is located on distal chromosome 1 in a region where bQTLs had been identified for locomotor activity, alcohol and pentobarbital withdrawal, open-field emotionality, and certain aspects of fear-conditioned behavior. This study was unable to determine whether or not the elements regulating *Kcnj9* expression were located within the bQTL intervals and/or near the gene locus. However, it is possible to make such links by combining gene expression and genotype data. Jansen and Nap (30) termed this approach “genetical genomics.” This approach was quickly adopted to examine gene expression in *Arabidopsis, Drosophila*, yeast, and the mouse [see (31) and references therein]. The expression QTLs (eQTLs) can be classified as either cis (mapping near the gene locus) or trans (mapping elsewhere in the genome) (32). When bQTLs and cis-eQTLs overlap, the cis-eQTL genes are inferred as causal genes [see e.g., (32)].

This general strategy from the perspective of HS populations has evolved in several important ways. First and beginning with Talbot et al. (33), mapping QTLs, including eQTLs in advanced HS populations has become relatively straightforward. QTL intervals of 1–2 Mbp can be routinely obtained and a haplotype signature for each QTL can be extracted. Behavioral and gene expression data are generally available for the founder strains, which facilitates the mapping process. Rather than using relatively expensive microarrays, very cost effective genotype information can now be obtained by low density genome-wide sequencing, which builds upon the detailed founder strain sequence information. Second, mouse microarrays used probes based on B6 sequence. Because of hybridization errors, this was problematic even for di-allele crosses and resulted in false positive eQTLs [see e.g., (34)]. For HS populations, the hybridization artifacts increase dramatically. RNA-Seq essentially solved this problem. However, RNA-Seq has its own set of problems and biases, which have been detailed elsewhere [e.g., (4, 35)]. Importantly for expression analysis in HS populations, alignment errors can occur. Although most RNA-Seq experiments use polyA+ RNA libraries, ribosome depleted RNA libraries can be used to also look at the expression of non-coding RNAs. Third, regardless of whether one uses microarrays or RNA-Seq for genome-wide studies, one is making thousands of comparisons. The number of independent comparisons is fewer than the number of genes detected since gene expression can be collapsed into modules with similar expression patterns. Perhaps the most widely used algorithm to detect these modules is the Weighted Gene Co-expression Network Analysis (WGCNA) (36), although there are many others. In the WGCNA, the general procedure is to extract the module eigengene (first principal component) and determine how well the eigengene aligns with the phenotype of interest. Since the number of modules formed is generally relatively small (e.g., 30–40), the multiple comparison penalty is greatly reduced. This approach is relevant to HS animals for at least two reasons. One, given that RNA-Seq is the preferred technology to analyze gene expression in HS populations, it should be noted that because of the difference in variance structure (compared to microarray data), RNA-Seq datasets have an advantage when constructing co-expression networks (37). Two, the expression variances in HS animals are higher than those found for diallel crosses of laboratory mouse strains (37). Although it may seem superficially counter-intuitive, increased variance will, up to a point, improve co-expression detection. Finally we note that the network based approaches allow one to differentiate hub and leaf nodes. Module hub nodes are generally defined as those in the top 10–20 percent of module connectivity, while the leaf nodes are those that collectively contribute the bottom 10 percent of connectivity.

Although not explicitly stated in the argument for developing the CC (22), one could imagine that by including the 3 wild-derived strains, splicing complexity would greatly increase. Related arguments could be used for developing any HS population. Zheng et al. (38) examined the splicing issue with paired-end sequencing (>160,000,000 reads/strain) of the ventral striatum in the 8 CC founder strains. Mapped junctions were >360,000 for all strains; but only 50% of these junctions were annotated. Strain specific splicing (SSS) events were those detected in only one strain. Sixty-four thousand strain-specific junctions were identified when all junctions were considered; however, for junctions with ≥3 or ≥10 read coverage, the numbers dropped to an average of ~3,000 and 500, respectively. The wild-derived strains, CAST/EiJ and PWK/PhJ, were demonstrated to have the highest percentages of strain-specific junctions. Some of these junctions were confirmed using qPCR. From the perspective of genetic diversity and splicing, this study should be seen as a starting point. The read density would likely need to be an order of magnitude higher to reliably detect rare splice junctions and rare SSS events. Further, any survey would need to include multiple brain regions.

## HS4 Mice and Multiple-Cross Mapping

We briefly introduce here the HS4, a relatively short-lived HS population (2001–2011). The HS4 was formed by crossing the B6, D2, BALB/cJ, and LP/J strains. Breeding details for the HS4 are found in Iancu et al. (24). It is important to note that a HS derived from only 4 strains can easily be completely balanced, while for an 8-way cross this is practically impossible (see above). A comparison of eQTL mapping in a B6xD2 F_2_, the HS4 and HS-CC is found in Iancu et al. (24). Two analysis methods were compared: HAPPY (39) and EMMA (40); the methods were also combined to produce joint method (JM). Single-marker (SM) QTL analysis tests for association between genotype at individual markers and the phenotype of interest here, gene expression. EMMA implements a variant of SM analysis. One essential feature of EMMA is to efficiently control for sample relatedness. HAPPY integrates information from several markers, and estimates the probability of descent from each of the founder strains and evaluates if there are significant phenotype differences between alleles inherited from the different progenitor strains. Perhaps the key observation from these analyses was the superior performance of the HS4 for detecting both *cis* and *trans* effects on gene expression when compared to the F_2_ and HS-CC. The superior performance was true regardless of the method used.

The HS4 were part of a project we and others termed multiple cross mapping (MCM). A summary of this project is found in Hitzemann et al. (41). Our interest in MCM was triggered by the observation that an open-field activity QTL was independently detected in three different mouse F_2_ intercrosses (B6xD2; B6xA; B6xBALB/c) (42–44); however, the QTL was not detected in D2xBALB/c or D2xLP crosses (45). We proposed that the information detected from multiple crosses could be used to sort microsatellite or SNP markers in order to detect chromosomal regions with the highest probability of containing QTLs. Empirically, the data described above suggested that there must be a region or regions on chromosome 1 where three strains (i.e., D2, BALB/c and A strains) are identical and different from the B6. Given that it was not possible to easily sequence the region(s) of interest, MCM appeared to be a reasonable approach. The down-side of MCM was that each cross required several hundred animals to be phenotyped and genotyped, whereas mapping in a HS would require fewer animals and provide greater precision [see Figure 15 in (41)]. With the advent of sequence data for the inbred mouse strains (21) and improvements in genotyping technology, including reduced costs, the MCM approach was no longer appropriate or necessary.

## High Drinking in the Dark (HDID) Selected Lines

Rhodes et al. (46) introduced the Drinking-In-the Dark (DID) procedure as a simple model of ethanol drinking to intoxicating blood ethanol concentrations (BECs). B6 mice regularly drank to BECs >1 mg/ml. Subsequently (3), DID was examined in a panel of inbred strains that included the 8 HS/NPT founders. The highest BECs (4 h DID trial) were obtained in the B6 and BALB/c strains, with males having somewhat higher BECs than females, even though females consumed higher amounts of ethanol. For all strains, the relationship between consumption and BECs was at best complex. Crabbe et al. (47) reported on the selection of the HDID-1 line; HS/NPT mice were the founders. The selection phenotype was the BEC at the end of a 4 h DID trial from the ingestion of a 20% ethanol solution. After 11 generations of selection, the BEC increased from 0.30 to 1.07 mg/ml. A replicate HDID selection (HDID-2) followed the HDID-1 selection. The lines were separated by 7 generations of breeding, but the selection response was largely identical [see Figure 3 in (48)]. Interestingly, the microstructure of drinking in the HDID-1 and -2 lines is different. HDID-1 mice drink in larger ethanol bouts than the HS founders, whereas HDID-2 mice drink in more frequent bouts (49). The observation that the two HDID lines appear superficially phenotypically similar but do show important differences is not unique to these replicate lines [see e.g., (50)]. In general, this should not be unexpected for a complex trait where no genes have a very large effect and where different allelic combinations can lead to a largely similar phenotype.

An issue we have indirectly addressed over the past few years is whether the transcriptional profiles associated with DID and ethanol preference overlap. Related to this issue, when compared across panels of inbred strains, DID and 2-bottle choice preference consumption appear to show some genetic overlap (3). Crabbe et al. (51) examined this issue in greater detail by looking at preference consumption in HDID-1 and the founder HS/NPT mice. The conclusion reached was similar; preference and DID consumption showed some genetic overlap, but this depended on the assay being used.

## HDID Selected Lines and Gene Expression

Iancu et al. (52) used the Illumina Mouse 8.2 array to examine gene expression in HDID-1, HDID-2, and HS/NPT mice (*N* = 48/group balanced for sex). An early version of the Mouse Universal Genotyping Array (MUGA) was used for genotyping; the MUGA contained 7,851 SNP markers, with an average spacing of 325 ± 191 kb. After elimination of non-polymorphic or low frequency (below 2.5%) SNPs, the data contained 3,683 markers further analyzed using a marker by marker approach (53, 54). The genotype data extracted (Figure 1) illustrated two important points. One, compared to the HS/NPT founders, genetic variance was strikingly reduced in both of the selected lines, presumably the result of the inbreeding that occurs when using a relatively small number of families for selection. Two, the genotype data illustrated that the selected lines were genetically distinct. The QTL analysis confirmed this point. Five unique QTLs exceeding the adjusted LOD threshold of 10.6 were found in the HDID-1 line and three unique QTLs were found in the HDID-2 line. There were however, three common QTLs on chromosomes 4, 14, and 16, each of which were mapped to relatively good (<5 Mbp) resolution. Of relevance to subsequent discussions, the Chr 14 QTL contained only 1 gene, protocadherin 17; the haplotype signature of the QTL corresponds to the LP/J strain being different than the other 7 founder strains. The QTL on Chr 4 has a similar haplotype (LP/J different from other founders) and a similar position and haplotype to a startle response QTL reported previously (19).

The gene expression analyses reported in Iancu et al. (52) and especially the integration of the differential expression and network analyses, set a pattern that has been repeated in our subsequent studies. The DE genes are in general, poorly connected to the co-expression network; i.e., the DE genes are largely leaf nodes. This cannot be unexpected. Unless the change in expression is very large, to detect DE the variance must be low. In contrast, construction of the co-expression network depends on a *robust* but biologically relevant variance structure. There were marked differences between the HDID-1 vs. HS/NPT and HDID-2 vs. HS/NPT in terms of the number of DE (FDR < 0.1) transcripts (1,430 vs. 301). One hundred and four transcripts were differentially expressed in both comparisons; 94 of these had the same directionality. A majority of the DE transcripts (85 out of 94) were found among the gray-network module, which is reserved for the poorly connected transcripts. GO annotation of the DE genes revealed significant enrichments in extracellular region part (*p* < 2 × 10^−3^) and the extracellular matrix (*p* < 5 × 10^−3^).

**Figure 2:**
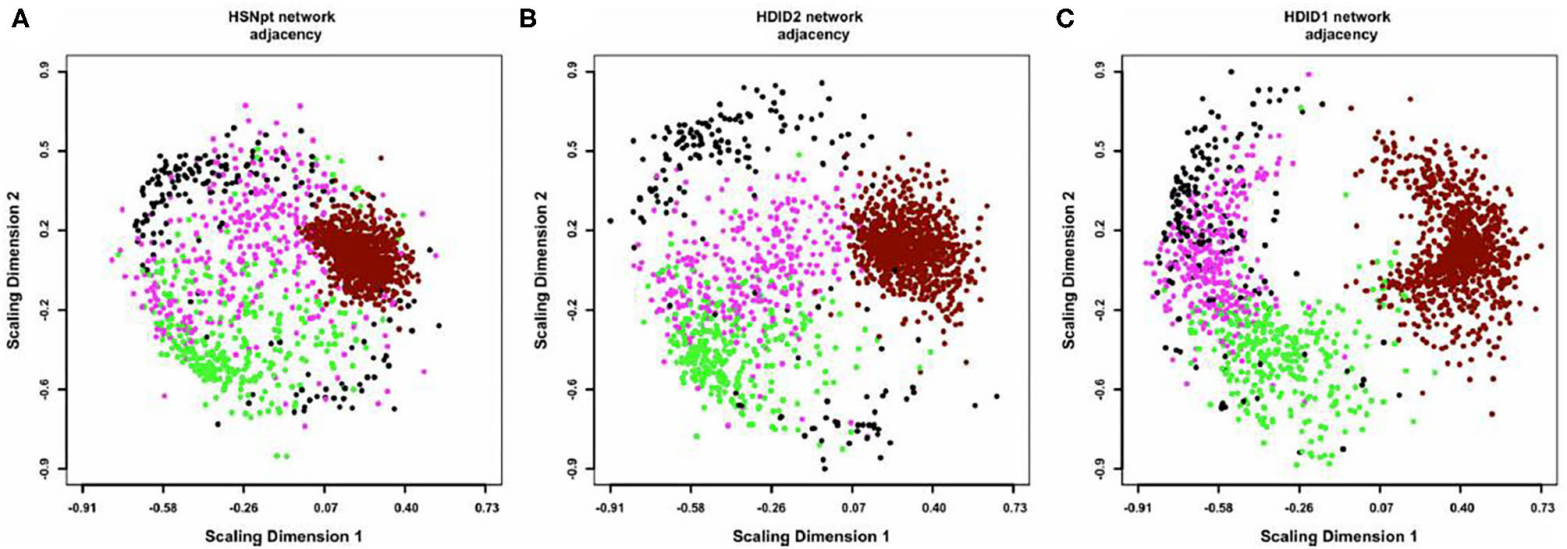
Multidimensional scaling (MDS) plots of the coexpression networks in HS/NPT **(A)**, HDID-2 **(B)**, and HDID-1 **(C)** datasets. For visual clarity, only the 4 modules most consistently affected by selection (“black,” “magenta,” “dark-red,” and “green”) are depicted. Each dot represents a transcript, with colors corresponding to module assignments. The distances between points correspond to network adjacency. The figure illustrates (1) the modularity of the networks, with similar colors clustered together and (2) the effect of selection on the network structure, with HDID-1,2 diverging from the original HS/NPT network structure. In particular, the “dark-red” module appears more dispersed, while the “magenta” module appears more compacted in the selection networks. Figure reprinted with permission from (52).

**Figure 3:**
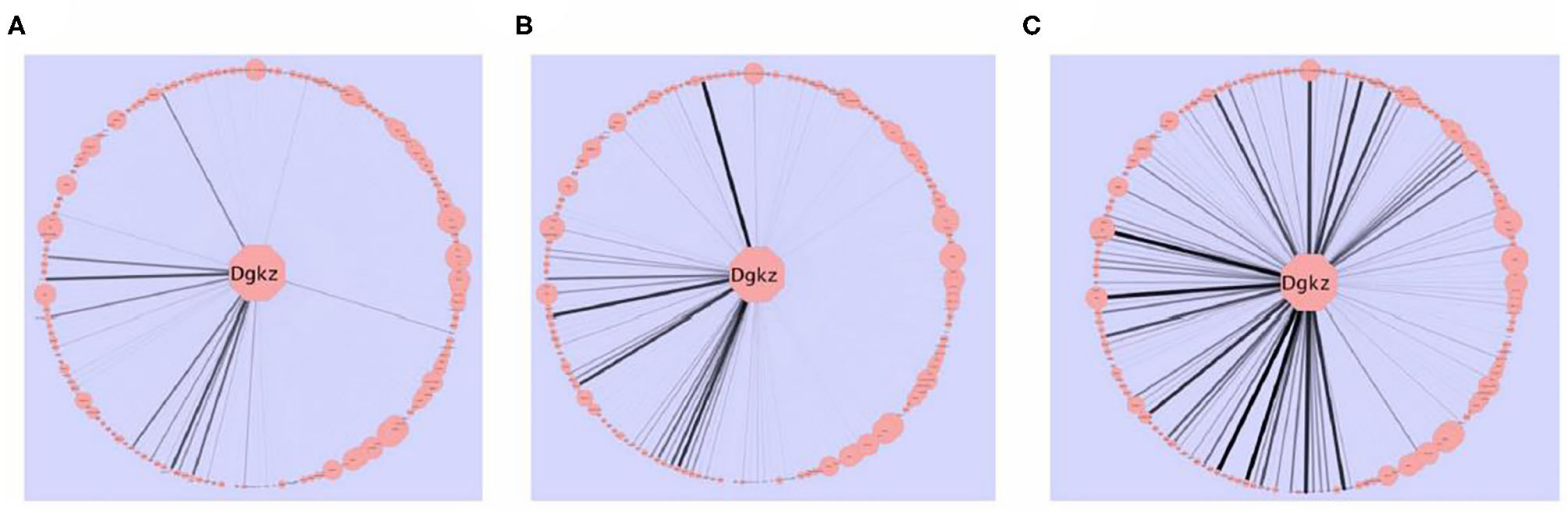
The effects of selection on intra-modular connectivity for *Dgkz*. *Dgkz* is found in the “black” module. Edge thickness and opacity are proportional with network adjacency between *Dgkz* and other module transcripts. The intra-modular connectivity of the other module genes is reflected in the node size. **(A)** HS/NPT network connectivity. **(B)** HDID-2 network connectivity. **(C)** HDID-1 network connectivity. Note the more pronounced increase in connectivity in the HDID-1 as compared to the HDID-2 animals. Figure reprinted with permission from (52).

A consensus network approach (55) was used to evaluate the effects of selection on transcriptome organization. Based on previous empirical observations (25), we concluded that in order to form modules of very high quality, sample sizes of ~ >40 are required [see Supplemental Table 2 in (52)]. With modules of high quality, module disruption is relatively easy to detect (module disruption may be either a significant increase or decrease in module connectivity). Separate networks were formed using the HS/NPT and each HDID line's expression data; differences between these networks were evaluated against random changes. An empirical distribution of random changes was generated by constructing networks (*N* = 1,000) using a mixture of samples from the HS/NPT and HDID animals. Bootstrapping and statistical significance assessment was performed over samples. Despite the genetic differences noted above, two of the co-expression modules (black and magenta [color has no meaning]) were similarly affected; i.e., the modules were significantly disrupted (see Figure 2). Both modules were highly enriched in neuronal genes (black module—*p* < 3 × 10^−27^; magenta module—*p* < 3 × 10^−5^). GO annotation of the black module revealed significant enrichments in neurological system process (*p* < 5 × 10^−6^), glutamate secretion (*p* < 7 × 10^−5^), and neurotransmitter transport (*p* < 8 × 10^−5^). GO annotation of the magenta module revealed significant enrichments in neuropeptide hormone activity (*p* < 2 × 10^−5^), peptide receptor activity (*p* < 9 × 10^−5^), and post-synaptic membrane (*p* < 2 × 10^−4^). The progressive effects of selection on *Dgkz*, a gene found in the black module and known to be associated with glutamate neurotoxicity and brain trauma, are illustrated in Figure 3. Gene module connectivity was increased in the HDID-2 animals and further increased in the HDID-1. Examples of selected genes in the magenta module and significantly affected by selection are found in Table 1 in (52). We bring two points to the readers' attention. The first is that both selections have affected a subgroup of GABA and glutamate related genes; this will be a familiar observation. The second point is the observation that selection affected the neuropeptide Y system. Manipulation of the neuropeptide Y system affects both DID and ethanol preference consumption [see (56) and references therein]. There is some evidence, at least for ethanol preference that these effects may be genotype-dependent (57).

Hoffman et al. (58) is the brain gene expression study focusing on ethanol preference that appears to be closest to Iancu et al. (52). Gene expression in HAP3 and LAP3 animals derived from HS/Ibg mice (59) were analyzed using Affymetrix microarrays. Although the analysis strategies were different, there appears to be no overlap of the DE genes detected in Iancu et al. (52).

Following on Iancu et al. (52), Iancu et al. (60) used RNA-Seq to compare the ventral striatal transcriptome of ethanol naïve HDID-2 mice and HS/NPT founders. Sample sizes were sufficient to analyze the male and female data separately. For females, the number of DE (FDR < 0.05) genes was 227; there was no significant GO enrichment for this grouping. For males, there were 1,525 DE genes, 836 and 689 genes were down- and up-regulated; 153 genes overlapped with the female grouping. Analysis of the down-regulated genes revealed significant enrichment in genes associated with extracellular matrix (ECM) organization and immune system process. No significant GO enrichment categories were detected for the up-regulated genes.

Beginning with Colville et al. (61) (see below), we introduced the differential variability (DV) metric into our analysis strategy [see (62–64)]. This computationally simple procedure identifies those genes that are likely to show a change in network connectivity. “For the DV metric, selection significantly (FDR < 0.05) increased the variability of 1,498 female genes and 766 male genes; 82 genes overlapped. Included in the overlapping subset were *Calb2, Gabrq, Nos1ap, Oxt, Pomc, Pvab, Slc6a11, and Trh*. For female genes with increased variance (*N* = 1,418), there was significant enrichment in annotations that included extracellular space, plasma membrane part, signaling receptor activity, and extracellular matrix organization. For female genes with decreased variance (*N* = 80), significant enrichment was detected for cytoskeleton of presynaptic active zone and axon part; genes involved included *Bsn, Pclo, Syn1, Myoc, Nav1, Tubb4a, Cplx2, and Ank3*. For male genes with increased variance (*N* = 663), there were significant enrichments in GO categories that included modulation of synaptic transmission, voltage gated cation channel activity, plasma membrane part, and synapse part. Genes in the latter category included *Grin2a, Grin2b, Dlg4, Gabbr2, Grm2, Pdyn, Gabra1, and Camk2a*. For male genes with decreased variance (*N* = 103), there were significant enrichments in GO categories associated with biological adhesion and extracellular part. From the perspective of the DV metric, which is closely aligned with network connectivity, the female and male data were largely mirror images” (64).

Additional analyses of this data set are found in Iancu et al. (60). However, the main observations are noted above. Two of these observations we wish to emphasize. The first is the involvement of neuroimmune systems, at least in males, in the DID phenotype. These data are consistent with the neuroimmune hypothesis of alcohol use disorder (AUD) (65). Sex differences in the alcohol-induced neuroimmune signaling are discussed elsewhere (64). The second point of emphasis are the data pointing to the involvement of the ECM. Alcohol and other drugs of abuse can have marked effects on ECM constituents [reviewed in (66–68)]. Ethanol has been shown to affect the brain expression of *tPA* (or *Plat*) (69, 70), *Mmp-9* (71), *Bcan* & *Ncan* (72), and *Tsp2* & *Tsp4* (73). Some data show that all elements of the brain ECM—the basement membrane, the interstitial ECM and the perineuronal nets– are affected by acute and/or chronic ethanol treatment (67). The evidence that changes in the brain ECM are associated with the *risk* for developing an AUD are less compelling. However, polymorphisms have been detected in *Mmp-9m, Tnc* & *Tnr* in human alcoholics (74, 75). Genome-wide association studies (GWAS) have revealed a polymorphism in *Col6a3* associated with alcoholism (76). Our data illustrate that HDID risk is associated with ECM associated genes in both males and females.

A common observation in both basic science and clinical populations is that substantial individual variation is retained even in groups at high risk for excessive ethanol consumption. Interestingly, this individual variation is seen even within inbred mouse strains such as the B6 [see (77)]. We asked whether the genes associated with individual variation in HDID-1 mice are different from those associated with selection (risk) (78). Thirty-five HDID-1 mice (18 males and 17 females) phenotyped for their BECs at the end of a standard 4-day DID trial, were sacrificed 3 weeks later. RNA-Seq was used to analyze the striatal transcriptome. Pearson correlations were used to assess the relationships between gene expression and the BEC. Five hundred and fifty-seven genes (375 positive vs. 182 negative) met the criteria for inclusion in the gene set enrichment analysis. The most significant (FDR < 0.01) annotation enrichments were for the positively correlated genes [Table 2 in (78)]. Broadly, the enriched gene categories were associated with the regulation of synaptic function. Genes associated with the category included *Grik5, Syn1, Stxbp1, Stx1a, Rims4, Rims1*, and *Stx1b Camk2g, Chrm3, Crhbp, Gria3, Grin1, Strn4, Syngap1* and *Syt2*. These data generally differ from those reported by Mulligan et al. (77) for individual DID variation in B6 mice. Given the differences in experimental design, such differences cannot be unexpected. However, perhaps their most salient conclusion is consistent with our results. “One hypothesis that evolved from our modular network analysis is that striatal medium spiny neurons may react to acute alcohol consumption with transcriptional changes that may underlie subsequent changes in behavior, including alcohol preference, tolerance and dependence” (77).

## HS-CC and Ethanol Preference

HDID selection has only used HS/NPT founders. Thus, there is no way to actually know if a different and/or simpler founder cross would yield similar results. However, for ethanol preference, we do have data that gets very close to this issue (see below). For those unfamiliar with alcohol preference research, selection from B6xD2 intercross animals and/or data collected from BXD RI strains has yielded remarkably consistent preference results for almost 30 years [see e.g., (79–81)].

Colville et al. (82) examined the transcriptional changes across three brain regions associated with selection for ethanol preference (24h/7d, 10% ethanol vs. water) from HS-CC founders. The three brain regions examined were the nucleus accumbens shell, the prelimbic cortex, and the central nucleus of the amygdala (CeA). Sample sizes were moderate (*N* = ~30/region/line). The selection protocol was short-term, terminated after four generations of selection. In the “High” line, ethanol preference more than doubled to ~0.5 whereas in the “Low” line preference was <0.1. As expected [see (83)] there were a large number of transcriptional changes, unique to each brain region. Here we focus on the changes that were common to all three regions [see Figure 3 in (82)]. *5730455P16Rik, Gdi2, Skiv2, Tsr1, and Glod4* were the only common DE genes. There were 30 common DV genes and this grouping was significantly enriched in genes associated with cell to cell signaling. Genes with this GO annotation included *Dlg2, Egr3, Gabbr2, Lnpep, Pcdhgb2, Pcdhac2, Sstr4, and Syt10*. The common DV genes were enriched in a common network module that differed in size across the three regions but shared common annotations. The three modules also shared 183 common genes. These common genes included several receptors; *Adra1a, Chrna7, Grin2b, Htr2a, Oprd1*, and *Sstr4*; 17 protocadherins including 14 of the 22 known protocadherins. Common hub nodes across regions included *Dlg2, Gatad2b, Pcdhac2, Tnks, Usp29*, and *Usp9x*.

**Figure 4:**
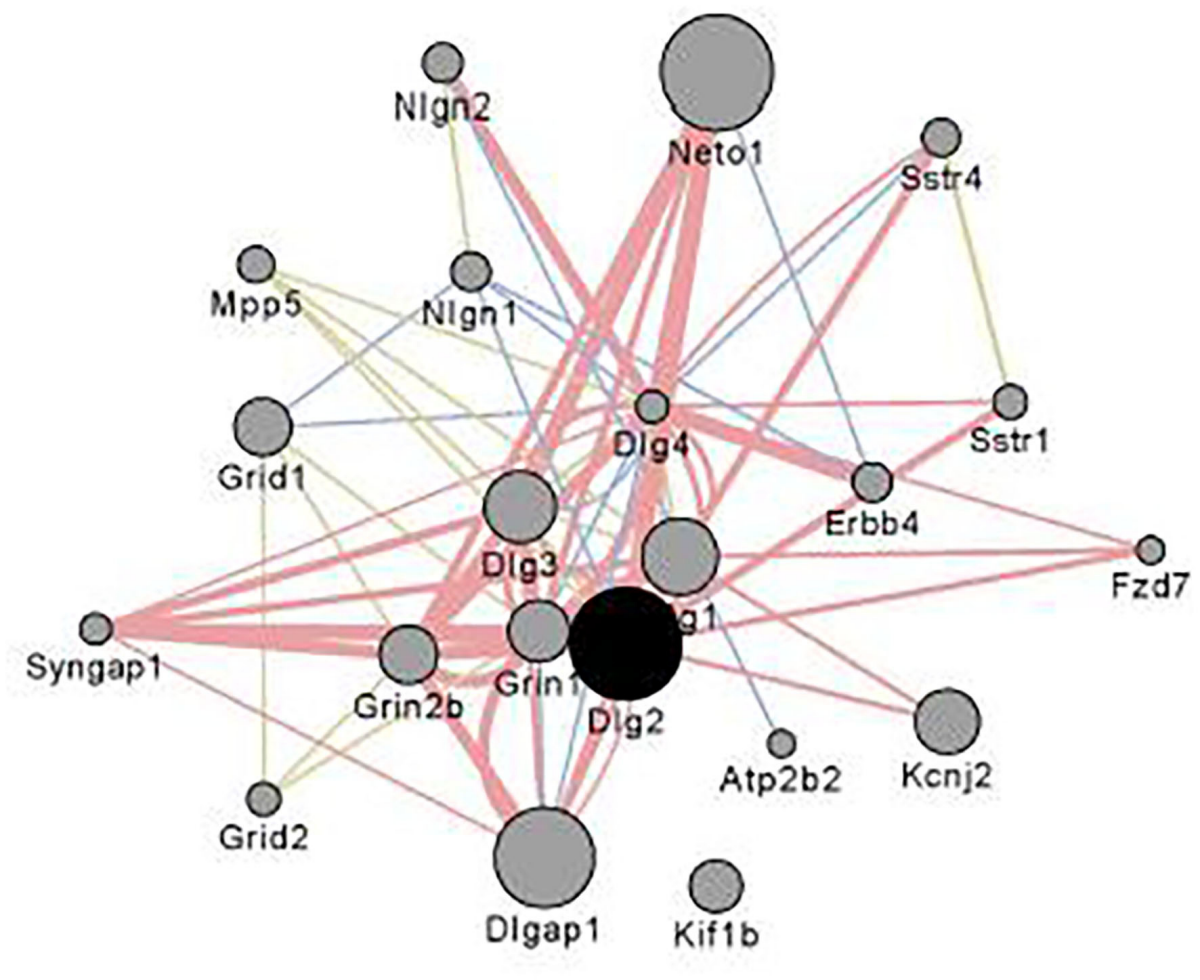
Interaction partners for *Dlg2* extracted using Gene Mania (84) which was accessed as a Cytoscape plugin with default settings. Depicted are top 20 genes related to *Dlg2* through physical interactions, colocalizations, or sharing protein domains. *Dlg2* which encodes for PSD93, interacts with a number of genes and gene products associated with glutamate receptor activity including *Dlg4, Syngap1, Neto, Grin1, Grin2b,Dlgap1* & *Dlg3*. Figure reprinted with permission from (82).

Figure 4 illustrates the coexpression and physical interaction partners for *Dlg2*. Key partners include a number of glutamate related genes: e.g., *Grin2b, Grid1 Dlg1, Dlg4*, and *Dlgap1*. These data extend the observations of Bell et al. (85) who noted when comparing ethanol naïve P and NP rats, there were a number of differences in glutamate signaling genes. Further, clinical studies have shown that in family history positive (FHP) individuals there is an altered response to the NMDA antagonist ketamine (86, 87).

A statistic added in Colville et al. (82) was differential wiring (DW). DW was restricted to search for Pearson correlations between individual genes that differed by >0.5. This general procedure has been used to quantify network rewiring in both genomic (88) and neural imaging studies (89). We identified for each gene, the number of changed edges and then inquired as to whether some genes had a disproportionately high number of changed edges. For the latter, a binomial test was used to test for significance. There were 72 significant DW genes common to all three brain regions and this grouping included *Chrna7, Als2, Pppir9a, Strn, Kcna4, Kif1a, and Slc1a2*. *Slc1a2*, which encodes for the excitatory amino acid transporter 2 (EAAT2); the inhibition of EAAT2 has been reported to reduce ethanol consumption (90).

Keeping the Colville et al. (61, 82) data in perspective, we turn to Kozell et al. (81). Beginning with a B6xD2 F2 intercross founder population, these authors selectively bred for both high alcohol consumption and low acute withdrawal (SOT line), or vice versa (NOT line). SOT is Old-English for habitual high alcohol user. Using randomly chosen fourth selected generation (S4) mice, RNA-Seq was employed to assess transcriptional differences in the ventral striatum between the SOT and NOT mice. Data were analyzed as described in Colville et al. (61, 82). For genes more highly expressed in the SOT line there was enrichment in genes associated with cell adhesion and post-synaptic membrane. The cell adhesion genes included 23 protocadherins, *Mpdz* & *Dlg2*. The post-synaptic membrane genes included *Gabrb3, Gphn, Grid1, Grin2b, and Grin2c* & *Grm3*. Thus, the SOT selection (81) and High preference line selection show overlapping transcriptional signatures. In contrast, the NOT line was enriched in genes with mitochondrial function.

The final study to be reviewed is Hitzemann et al. (68), which examined in HS-CC mice the effects of chronic (13 weeks) ethanol consumption [24h/7d 2-bottle choice] on CeA gene expression. Here we focus on the correlation of individual gene expression and week 13 ethanol preference. For females, the enriched annotations associated with cilium organization, extracellular region, and collagen-containing ECM. For males there were no significant annotation enrichments.

The majority (70%) of female genes correlated with preference were found in a single WGCNA network module. This module was enriched (*p* < 0.0001) in genes with an astrocyte annotation and in annotations associated with the extracellular matrix and cilium. Among the female genes positively correlated with preference, 43 were top hub nodes. “Enrichr (91, 92) was used to search for key transcription factors among the top hub nodes. A key finding was that 19 of the top nodes were down-regulated in an orthodenticle homeobox 2 (*Otx2*) knockout mouse [GSE27630; (93)]. *Otx2* is often referred to as a master regulator, and known to have key roles in brain patterning and post-natal plasticity. *Otx2* is further required for generation of various neuronal subpopulations, including ocular motor and midbrain dopaminergic neurons (94, 95), and development and maintenance of perineuronal nets. In the adult brain, *Otx2* expression is largely localized to the choroid plexus (96). The OTX2 protein is captured by the perineuronal nets and accumulated in parvalbumin type GABA-ergic neurons throughout the brain (97). Our data indicate a low, but detectable expression of *Otx2* in the CeA, affected by ethanol exposure and predicted to have a role in the escalation of ethanol preference seen in HS-CC females, but not males, and in the observed sex differences in the transcriptional response” (68). Of related interest, Coles and Lasek (98) found that DID increased *Otx2* expression in the VTA; however, viral mediated down-regulation of *Otx2* did not affect ethanol consumption.

## Discussion

For more than 50 years, HS and other outbred rodent populations have been key to investigating the genetics and basic biology of ethanol phenotypes, including excessive ethanol consumption. To put the current use of HS animals in perspective it is useful to return to Gora-Maslek et al. (99) who observed that a panel of BXD RI strains, even with a sparse genetic map, could be used to map drug-related QTLs. However, this study also illustrated a point that continues to complicate genomic research: gene effect sizes for essentially all complex traits are very small. To confirm a BXD generated QTL with an effect size of 5 percent (actually a very large effect!) would require ~600 B6xD2 F_2_ intercross animals. While confirmation was possible, resolution of the QTL was poor, given the relatively low number of recombinations in the F_2_ population. One suggested solution to this problem was to generate from the F_2_ an advanced intercross that would build the recombination density [see e.g., (100)]. This solution introduced a new problem. Since it is practically impossible to generate an advanced intercross with a very large number of families, substantial relationships among individuals will develop over time and relatedness becomes a confounding factor. HS animals and selected lines have this same problem. As noted above, there are algorithms that deal with relatedness and importantly these are included in recent updates to r/QTL (101). Regardless of how one deals with the relatedness issue(s), it would seem that independent replication should be a convincing solution to the problem. For QTLs associated with ethanol preference and derived from B6xD2 crosses, replication has worked extremely well (79–81). However, replication in HS animals does not appear to be straightforward. As shown in Iancu et al. (52), the replicate HDID-1 and HDID-2 selections yielded only partially overlapping QTL results. These data suggest that with the increase in genetic diversity, different sets of genes can be employed to produce a similar phenotype, in this case high BECs. In addition, detailed analysis of the drinking behavior in the two selected lines revealed that there are differences—one favors larger bouts and the other favors more bouts to increase BECs. From a certain perspective, one could argue that these differences in genotype and phenotype are precisely the reasons one uses an HS population, to generate a diversity of results, detecting new pathways and mechanisms of action. However, one can also understand why this diversity is not universally appealing.

The argument that new mechanisms will be revealed as genetic diversity increases has rarely been tested under identical laboratory conditions. As noted above, Iancu et al. (24) examined eQTL expression in the striatum of F_2_, HS4 and HS-CC animals. This experiment was conducted using Illumina microarrays; in order to prevent hybridization artifacts, any probe sequence known to contain a SNP from one of the founder strains, was removed from the analysis. As noted previously, the detection of *cis* and *trans* eQTLs was the most reliable in the HS4. However, the detection of *trans* eQTLs was higher in the HS-CC. However, interpretation of these data are complicated by the complex kinship matrices among samples, which differ on a chromosome by chromosome basis. The question naturally arises as to how these and other changes in the regulation of gene expression will affect issues such as selection for a behavioral phenotype and the associated transcriptional changes. The only data we have for an ethanol phenotype (ethanol preference) are described above and suggest that there is likely overlap between the F_2_ and HS-CC along dimensions related to glutamate synaptic transmission and cell adhesion. However, for a different phenotype, haloperidol-induced catalepsy, we have a very direct comparison among F_2_, HS4 and HS-CC animals (102). Haloperidol-induced catalepsy is highly heritable (*h*^2^ > 0.6), and the mechanism of action is well-known (blockade of D_2_ receptors), as is the target brain region (the striatum). Short-term selective breeding was used for all 3 populations and selection was stopped after 3 generations. The High and Low lines differed by 30 fold or more in the haloperidol ED_50_; the lines also differed in their response to raclopride and showed no difference in the response to the D_1_ antagonist, SKF23390. Microarrays were used to analyze gene expression. The number of differentially expressed transcripts (FDR < 0.1) was significantly higher in the HS-CC compared with the F_2_ and HS4 selections (445 vs. 113 and 33, respectively). There were no differentially expressed transcripts common to all 3 selections. A consensus network approach, previously described, was used to compare the effects of the 3 selections. A relatively large number of transcripts significantly changed network connectivity: 458 (7.0%), 499 (7.6%), and 1,537 (23.4%) in F_2_, HS4 and HS-CC populations, respectively. However, as for differential expression, none of the differentially connected transcripts were shared in common across the 3 selections. Our analysis revealed that, for each selection, several modules significantly (*Z* < −2) changed intra-modular connectivity structure: 4 modules in the F_2_, 12 in the HS4 and 21 in the HS-CC. There were 3 affected modules in common to all selections and in these the HS-CC showed the largest changes in connectivity. Importantly, and we believe this is the most *salient* point, there was no overlap among the 3 populations in the genes that showed a change in module connectivity. The common feature was the module(s) not the genes; the common modules were enriched in annotations associated with intracellular signaling and locomotor behavior. The latter category included *Drd2, Chat, and Pde10a* & *Rgs9*. These data suggest that combining the results from populations at different levels of genetic diversity could be key to finding new (or old) targets for therapeutic manipulation.

**Figure 5:**
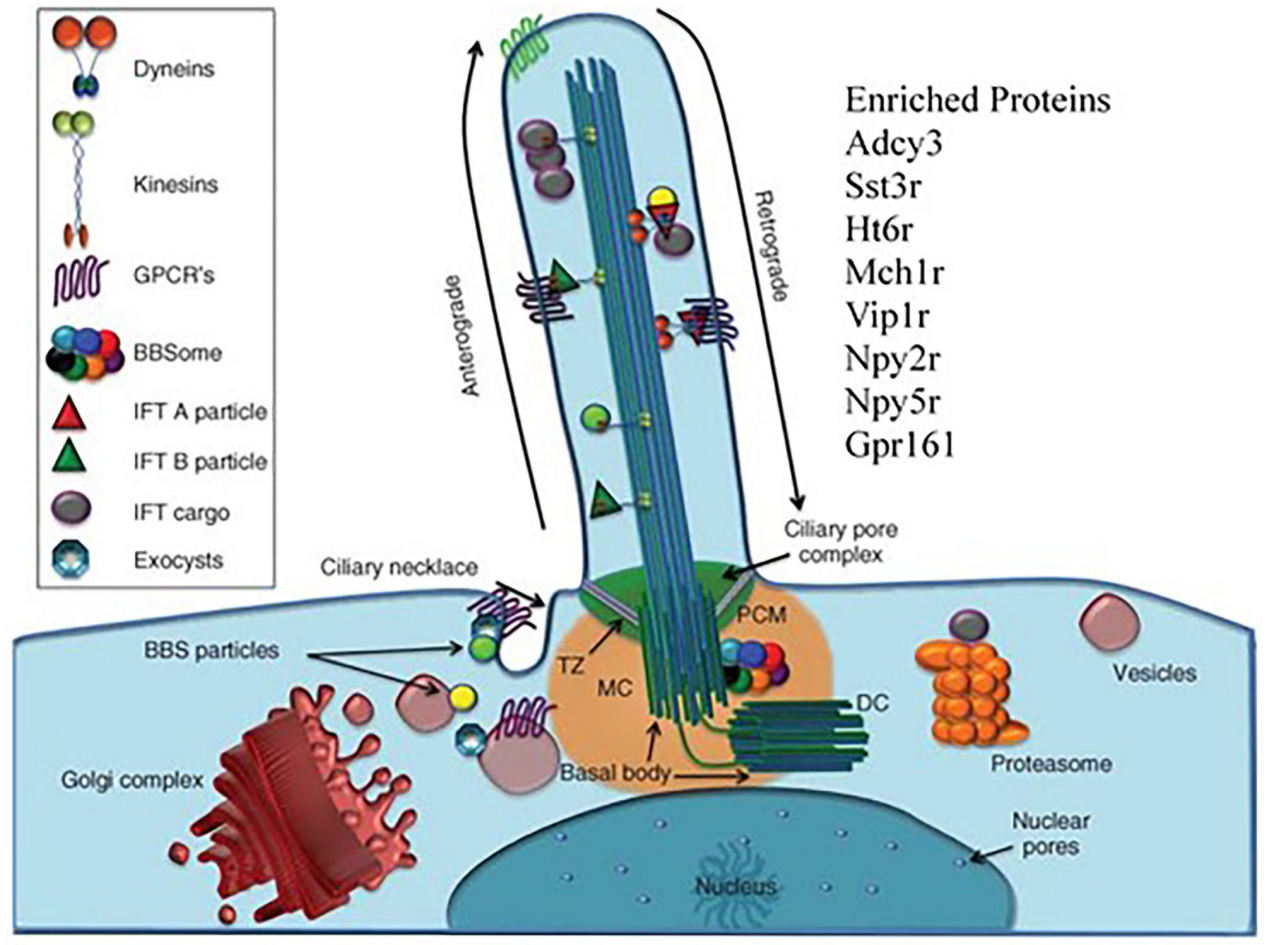
The primary cilia as target for the effects of chronic ethanol consumption. There are to our knowledge, no reports linking the brain's primary cilia to ethanol preference, chronic ethanol exposure or excessive ethanol consumption. Our data shows that there is a strong cilia signal associated with individual variation in preference within the context of chronic ethanol exposure. The primary cilium is a long, thin organelle protruding from the apical surface of almost all cell types. This structure is formed when the cell is in G0 or G1 phase, and often during S/G2 phase (104). The timing of cilium formation, “ciliogenesis,” is restricted to these stages of the cell cycle because the cilium is rooted at its base by the basal body, which is derived from the mother centriole of the centrosome (105). Differently than mobile cilia, the axoneme of the primary cilium has a “9+0” structure and is not mobile. The “primary cilia” are fundamentally important for normal cell signaling during development and homeostasis, resulting in the adoption of the term “cell's antenna” (106). These signaling functions are carried out by the myriad of signaling molecules.

Our last point is that working with HS animals may be beneficial in finding the truly unexpected. We return to Hitzemann et al. (68) which focused on the transcriptional changes associated with a 13 week preference trial. The unexpected observation was that in females the transcriptional features associated with week 13 preference were enriched in cilium annotations. Alcohol is known to affect the motile cilia in the brain's ventricles and other tissues [see e.g., (103)]. However, in the CeA and other brain regions there will only be primary cilia in neurons and astrocytes. There are a number of proteins localized in the neuronal primary cilia (Figure 5). These include ADCY3, SSTR3, and HT6R. There is some evidence that the manipulation of these cilia-specific molecules affects ethanol consumption. For example, de Bruin et al. (107) found that a highly selective HT6R antagonist (CMP 42) attenuated both nicotine- and alcohol-seeking behaviors in Wistar rats. Further, *Ht6r* knockout mice are less sensitive to alcohol-induced ataxia and sedation (108), and HT6R antagonists reduce cocaine self-administration, attenuate cue-induced reinstatement, attenuate the expression of cocaine-induced conditioned place preference, and reduce the acquisition and expression of nicotine-induced sensitization [see references in (109)]. The orphan receptor, GPR88, is enriched in striatal neuronal primary cilia (110). The GPR88 agonist, RTI-13951-33, significantly reduces alcohol self-administration and intake in female Long-Evans rats in a dose-dependent manner, without effects on locomotion and sucrose self-administration. However, given that the module is enriched in astrocyte annotation genes, it could be reasonably argued that our attention should focus on astrocyte primary cilia. However, as noted by Sterpka and Chen (111), “Presently, little is known about the function, signaling pathways, and structural dynamics of astrocytic primary cilia in the mature brain, although astrocytes fulfill a wide range of functions including providing trophic support, maintaining homeostasis, and protecting neurons from acute insults or brain injury (112). Since astrocytes can proliferate under certain pathological conditions (113), astrocytic primary cilia are not static but subject to dynamic changes.”

For most of the past 50 years, the use of HS mice largely has been limited to selective breeding; several examples of this approach in the context of ethanol research have been described. However, given that all the founder strains of existing HS populations have been deeply sequenced, it is now possible to precisely map QTLs in HS mice in much the same way one uses a GWAS approach to map human QTLs. The founders of the HS-CC and DO populations possess ~50 million SNPs. Thus, it is likely that there are allelic variants associated with the expression of every gene. Further, there are no rare alleles; absent the effects of genetic drift, the minimum allele frequency in an 8-way cross is 12.5%. With rare exception, because behavioral traits of interest are complex and polygenic, with no one gene accounting for a large percentage of the genetically-determined variance, sample sizes need to be scaled accordingly. Unlike human studies, the environment for mouse studies can be strictly controlled or modified in ways to test specific hypotheses. For some human disorders such as schizophrenia or major depressive disorder, a relevant mouse model seems unlikely. This challenge is considerably lessened for AUDs and substance abuse disorders and it is for such conditions that we believe HS mice will serve an important role in detecting new mechanisms of action that will lead to the development of new therapeutic approaches.

## Author Contributions

RH wrote the first draft of the manuscript. All authors contributed to manuscript revision, read, and approved the submitted version.

## Funding

Funding sources were NIH NIAAA P60 AA 010760 (RH, AO, and TP), R01 AA 011034 (RH), U01 AA 013484 (RH), R24 AA020245 (RH and TP), U01 AA 013519 (AO), I01 BX 004699 (AO), and the Office of Research, Department of Veterans Affairs (AO and TP).

## Conflict of Interest

The authors declare that the research was conducted in the absence of any commercial or financial relationships that could be construed as a potential conflict of interest.

## Publisher's Note

All claims expressed in this article are solely those of the authors and do not necessarily represent those of their affiliated organizations, or those of the publisher, the editors and the reviewers. Any product that may be evaluated in this article, or claim that may be made by its manufacturer, is not guaranteed or endorsed by the publisher.
